# Temporal changes of the incidence of childhood B-cell precursor acute lymphoblastic leukaemia in Germany during the COVID-19 pandemic

**DOI:** 10.1038/s41375-022-01730-x

**Published:** 2022-10-26

**Authors:** Arndt Borkhardt, Joachim Schüz, Claudia Trübenbach, Maike Wellbrock, Claudia Spix, Friederike Erdmann

**Affiliations:** 1 Department of Paediatric Oncology, Haematology and Clinical Immunology, Center for Child and Adolescent Health, Medical Faculty, Heinrich-Heine-University, Moorenstraße 5, 40225 Düsseldorf, Germany; 2 German Cancer Consortium (DKTK), partnering site Essen/ Düsseldorf, Düsseldorf, Germany; 3 Environment and Lifestyle Epidemiology Branch, International Agency for Research on Cancer, World Health Organization (IARC/WHO), 150 cours Albert Thomas, 69372 Lyon, France; 4 Division of Childhood Cancer Epidemiology, Institute of Medical Biostatistics, Epidemiology and Informatics (IMBEI), University Medical Center of the Johannes Gutenberg University Mainz, Obere Zahlbacher Straße 69, 55131 Mainz, Germany; 5 Department of Prevention and Evaluation, Leibniz Institute for Prevention Research and Epidemiology—BIPS, Achterstraße 30, 28359 Bremen, Germany

**Keywords:** Leukaemia, Leukaemia, Epidemiology

Acute lymphoblastic leukaemia is the most common malignancy in children, but despite a growing body of research, its aetiology remains incompletely understood and there is a lack of primary preventive measures. The complex interplay between genetic predispositions—be it inherited or somatically acquired during fetal development—and postnatal environmental triggers has become a focus of research into childhood acute lymphoblastic leukaemia (ALL). There is evidence that specific patterns of exposure to common infectious agents may act as such a postnatal trigger [1].

In this regard, the SARS-CoV-2 pandemic and all the social and behavioural measures taken to counteract the spreading of the virus in the population may have influenced the incidence of those infections, particularly in children of 2–6 years with B-cell precursor ALL (BCP-ALL), the ALL subtype for which the association with infections has widely been proposed [2].

Exposure to viral challenges may favour the outgrowth of a pre-existing, pre-leukaemic clone. This supposition has been supported by the increased incidence of childhood ALL several months after influenza epidemics, infection-associated space-time clusters of BCP-ALL, and preclinical animal models [3–5]. Moreover, in some children clinical diagnoses of overt BCP-ALL were preceded by a SARS-CoV-2 infection [6]. It remains speculative, however, whether this observation reflects just coincidence or suggests a causative effect of the infectious trigger.

By contrast, the closing of day-care facilities, kindergartens and schools during the pandemic years may have lowered general viral exposure, but it may also have reduced the physiological training and maturation of children’s immune systems. As Greaves already pointed out, it would be very interesting to know how many children with BCP-ALL diagnosed in 2020 and beyond had previously been infected with SARS-CoV-2, and how this compares to the data for children with other types of leukaemias or solid tumours [7].

Those data are largely missing so far, but will likely be available in the future. In Germany, the nationwide, population-based assessment of the impact of SARS-CoV-2 on the incidence of all childhood cancers indicated a general increase in 2020 which persisted to some extent in 2021 (refs. [8, 9]). We previously speculated that an increase in awareness among parents and paediatricians during the pandemic might be the main contributor, since the marked increase was seen across all diagnostic groups and cancer types (and was not limited to ALL). On the other hand, as the increase varied somewhat (between 8% and 12%) across diagnostic groups and cancer types, other effects, including changes in underlying cancer risk, cannot be ruled out, including for BCP-ALL. Hence, we modelled the effect of a lack of immune training due to the closing of day-care facilities in Germany in terms of the respective increase of the at-risk population, and projected approximately 20 ALL excess cases per year among 2–6-year-olds [10]. This corresponds to an increase of 6.4%, which might be attributable to lockdown measures. In the present report, we provide the first BCP-ALL-specific incidence estimates for the years 2020 and 2021. We used the high-quality population-based data from the national German Childhood Cancer Registry (GCCR) and identified all incident diagnoses of BCP-ALL in 0–14-year-olds (defined according to group I(a)1 of the International Classification of Childhood Cancer, third edition (ICCC-3), precursor cell leukaemias, which may also include a few single cases of precursor cell leukaemias other than BCP) [11].

The GCCR registration process is based on daily reporting by all paediatric haematology-oncology units in Germany, with virtually complete registration [12]. For this study, we used the most up-to-date status of the GCCR database, including late reports received by 15 March 2022. We calculated age-standardized (ASR; using the Segi 1960 World Standard Population) [13] and age-specific incidence rates per 1 000 000 person-years. Due to the fact that BCP-ALL markedly peaks in 2–6 year-olds, we used two different age strata, namely ages 2–6 years and 7–14 years with the latter considered as comparison group. Incidence rates for 2021 were estimated by applying two different scenarios of late reporting (specified in Table 1).

**Table 1:** Estimated age-standardized and age-specific incidence rates of B-cell precursor acute lymphoblastic leukaemia in children aged 0–14 years in Germany in 2020 and 2021 (by applying different hypothetical scenarios of additional cases due to late reporting) in comparison to previous years.

	2005–2009		2010–2014		2015–2019^3^		2020^4^		2021 (SI)^5,6^		2021 (SII)^5,7^	
	*N* cases	ASR^1^ per 1 000 000 [95% CI]	*N* cases	ASR^1^ per 1 000 000 [95% CI]	*N* cases	ASR^1^ per 1 000 000 [95% CI]	*N* cases	ASR^1^ per 1 000 000 [95% CI]	*N* cases	ASR^1^ per 1 000 000 [95% CI]	*N* cases	ASR^1^ per 1 000 000 [95% CI]
B-cell precursor acute lymphoblastic leukaemia^2^	2308	44.1 [42.3–46.0]	2090	41.7 [40.0–43.6]	2225	42.2 [40.4–44.0]	512	46.4 [42.5–50.5]	481	43.9 [40.1–48.0]	482.9	44.1 [40.2–48.1]
	*N* cases	Age-specific incidence rate per 1 000 000 [95% CI]	*N* cases	Age-specific incidence rate per 1 000 000 [95% CI]	N cases	Age-specific incidence rate per 1 000 000 [95% CI]	*N* cases	Age-specific incidence rate per 1 000 000 [95% CI]	*N* cases	Age-specific incidence rate per 1 000 000 [95% CI]	*N* cases	Age-specific incidence rate per 1 000 000 [95% CI]
2–6 years of age	1316	72.7 [68.8–76.7]	1204	70.2 [66.3–74.2]	1329	72.2 [68.3–76.2]	312	79.1 [70.6–88.4]	269	68.2 [60.3–76.9]	269.9	68.5 [60.5–77.1]
7–14 years of age	763	23.8 [22.2–25.6]	674	22.5 [20.8–24.3]	669	22.8 [21.1–24.6]	145	24.4 [20.6–28.8]	140	23.6 [19.9–27.9]	140	23.6 [19.9–27.9]

^1^*ASR* age-standardised incidence rate (using Segi World Standard Population) per 1 000 000 person-years. Annual population numbers were obtained from the Federal Statistical Office. Since at time of analysis population numbers for 2021 were not yet available, we applied the population numbers of 2020 to 2021.

^2^Defined using group I(a)1 of the International Classification of Childhood Cancer, third edition (ICCC-3).

^3^Age-standardised and age-specific incidence rate per 1 000 000 person-years in 2015–2019. Incidence rates for 2015–2019 included all cases reported in the respective year or the subsequent year; cases reported only after the subsequent calendar year were not included.

^4^Age-standardised and age-specific incidence rate per 1 000 000 person-years in 2020. Incidence rates included all cases reported in the respective year or the subsequent year; cases reported only after the subsequent calendar year were not included.

^5^Although the GCCR receives information on newly diagnosed cases on a daily basis, some cases (5.8% of all BCP-ALL cases) of a calendar year are reported with some delay in the subsequent calendar year. For 2020, approximately 99.4% of all incident BCP-ALL cases of that year were reported by 15 March 2021. ASR and age-specific incidence rates for 2021 were estimated by applying two different hypothetical late-reporting scenarios.

^6^Scenario I: considering no additional cases due to late reporting after 15 March 2022.

^7^Scenario II: estimated age-standardised and age-specific incidence rate per 1 000 000 person-years in 2021, applying the proportion of additional cases due to late reporting after 15 March 2021 observed for incident diagnoses in 2020 (specific to age groups). The proportion of additional cases due to late reporting after the 15 March 2021 amounted to 0.39% for B-cell precursor acute lymphoblastic leukaemia overall, 1.85% for B-cell precursor acute lymphoblastic leukaemia at ages 0–1 years, 0.32% for B-cell precursor acute lymphoblastic leukaemia at ages 2–6 years and 0% for B-cell precursor acute lymphoblastic leukaemia at ages 7–14 years.

We observed a remarkable increase in the incidence in 2020 compared to the ASRs in previous years. In particular, the age-specific incidence rate in 2–6-year-olds increased by 9.6% to 79.1 cases per million (Table 1 and Fig. 1). This increase corresponded to 27.5 additional (absolute) cases in 2020 compared to the expected number averaged from the five previous years (2015–2019). Notably, an incidence rate as high as 79.1 for BCP-ALL in 2–6-year-olds has not been observed since the GCCR was established in 1980. However, this ASR peak in 2020 was followed by a remarkable regression in 2021, when the age-specific incidence rate in 2–6-year-olds dropped to 68.5 cases per million (Table 1). These temporal changes in the incidence rates were less pronounced in the 7–14-year-olds (increase of 7.0% vs 9.6% in 2–6-year-olds in 2021), where the same strict infection control measures had been similarly applied (Fig. 1); less pronounced but the difference being compatible with random variation. In contrast to our own projections, we observed little indications for an excess of BCP-ALL cases in 2021. We also did not observe a clear general reduction in ALL cases as was reported for the post-Sars-CoV-1 period in Hong Kong in 2003 ref. [14].

**Fig. 1:**
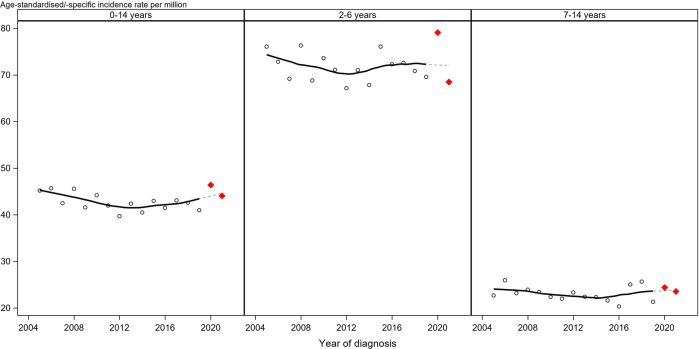
B-cell precursor acute lymphoblastic leukaemia (BCP-ALL) incidence rate fluctuations in 2–6 year-olds as compared to 7–14 year olds. Age-standardized (children aged 0–14 years; using Segi World Standard Population) and age-specific incidence rates (2–6/ 7–14 years) of B-cell precursor acute lymphoblastic leukaemia in Germany over time. Incidence rates for 2021 were estimated by adding the proportion of cases added to incident diagnoses in 2020 after 15 March 2021 due to late reporting (Scenario II, see Table 1). For graphical presentation, a locally estimated scatterplot smoothing (LOESS) with cubic interpolation was applied to the incidence rates of 2005–2021 by calendar year. Single incidence estimates for the years 2020 and 2021 are represented by red diamonds.

It should be kept in mind, however, that childhood BCP-ALL is a rare disease and thus epidemiological observations show considerable random variation and statistical uncertainty, as reflected in the wide and overlapping confidence intervals (see Table 1 and Supplemental Table 1). For this reason, the incidence estimates of the one-year age bins for 2020 and 2021 in comparison to previous years should be interpreted even more cautiously (Supplemental Table 1) than the estimates for the larger age groups but may serve as a valuable data source for future international comparisons. There are several additional limiting factors in interpreting BCP-ALL rates in relation to the pandemic. The degree of lockdown was not uniformly strict throughout Germany and the overall prevalence of (silent) SARS-CoV-2 infections in children with BCP-ALL remains unknown to date. Recent reports from Germany suggested a considerable rate of very young children (0–3 year-olds) who acquired clinically unrecognized SARS-CoV-2 infection and turned out to be seropositive [15].

Vaccination programmes against SARS-CoV-2 started in early 2021, but 2–6-year-olds were vaccinated only under very rare circumstances, e.g., a pre-existing underlying severe chronic disease or strong parental insistence.

In conclusion, we believe that monitoring incidence rates alone will not give the final answer, so serological screening for antibodies against SARS-CoV-2 in children with BCP-ALL as well as in a population-based comparison group remains highly recommended. The remarkable increase in 2020 followed by a regression in 2021 seen in 2–6-year olds appears to be too strong to be just random fluctuation. The slightly weaker effect in older children may support, albeit indirectly, the idea that viral infections as well as their prevention through social distancing, wearing facemasks, and lockdown measures may have two opposing effects on the development of the disease. The years to come will reveal whether the lessons learned during the SARS-COV-2 pandemic will pave the way for developing preventive strategies against this common childhood cancer.

## Supplementary information

Supplemental Table 1

## Data Availability

Under the permission that national data protection requirements are fully met, access to aggregated or pseudonymised individual-level data may be made available upon reasonable request. All data access requests should be directed to the German Childhood Cancer Registry.
