# Supplementary material for: Temporal changes of the incidence of childhood B-cell precursor acute lymphoblastic leukaemia in Germany during the COVID-19 pandemic
Source: Leukemia. 2022 Oct 26;36(12):2908–11. doi: 10.1038/s41375-022-01730-x (PMC9607786; doi:10.1038/s41375-022-01730-x)
Supplement: Supplementary file 1 — Supplemental Table 1 [file 41375_2022_1730_MOESM1_ESM.docx]

Supplemental Table 1: Estimated age-specific incidence rates of B-cell precursor acute lymphoblastic leukaemia^1^ in children aged 2 – 6 years by single years of age in Germany in 2020 and 2021 (by applying different hypothetical scenarios of additional cases due to late reporting) in comparison to previous years.

|  |  | |  | |  | |  | |  | |  | |
| --- | --- | --- | --- | --- | --- | --- | --- | --- | --- | --- | --- | --- |
|  | **2005-2009** | | **2010-2014** | | **2015-2019^2^** | | **2020^3^** | | **2021 (SI)^4,5^** | | **2021 (SII)^4,6^** | |
| **Age at diagnosis** | **N cases / N of individuals at risk (min; max)^7^** | **Age-specific incidence rate per million [95% CI]** | **N cases / N of individuals at risk (min; max)^7^** | **Age-specific incidence rate per million [95% CI]** | **N cases / N of individuals at risk (min; max)^7^** | **Age-specific incidence rate per million [95% CI]** | **N cases / N of individuals at risk (min; max)^7^** | **Age-specific incidence rate per million [95% CI]** | **N cases / N of individuals at risk (min; max)^7^** | **Age-specific incidence rate per million [95% CI]** | **N cases / N of individuals at risk (min; max)^7^** | **Age-specific incidence rate per million [95% CI]** |
| **2 years** | 343 /  3 491 123  (682 949; 717 264) | 98.2  [88.1-109.2] | 332 /  3 406 667  (671 677; 689 624) | 97.5  [87.3-108.5] | 348 /  3 790 498  (704 989; 802 533) | 91.8  [82.4-102.0] | 77 /  800 391 | 96.2  [75.9-120.2] | 98 /  800 391 | 122.4  [99.4-149.2] | 99.3 /  800 391 | 124.1  [100.9-151.0] |
| **3 years** | 381 /  3 541 254  (683 114; 733 025) | 107.6  [97.1-119.0] | 331 /  3 414 212  (673 178; 692 026) | 96.9  [86.8-108.0] | 375 /  3 723 055  (697 433; 792 290) | 100.7  [90.8-111.5] | 81 /  807 007 | 100.4  [79.7-124.8] | 55 /  807 007 | 68.2  [51.3-88.7] | 55 /  807 007 | 68.2  [51.3-88.7] |
| **4 years** | 290 /  3 615 313  (698 412; 758 513) | 80.2  [71.2-90.0] | 210 /  3 423 642  (678 225; 695 088) | 61.3  [53.3-70.2] | 258 /  3 665 652  (698 991; 774 387) | 70.4  [62.1-79.5] | 68 /  796 872 | 85.3  [66.3-108.2] | 60 /  796 872 | 75.3  [57.5-96.9] | 60 /  796 872 | 75.3  [57.5-96.9] |
| **5 years** | 194 /  3 691 380  (708 130; 777 013) | 52.6  [45.4-60.5] | 179/  3 446 302  (685 000; 698 950) | 51.9  [44.6-60.1] | 194 /  3 627 995  (702 357; 755 604) | 53.5  [46.2-61.6] | 52 /  778 617 | 66.8  [49.9-87.6] | 27 /  778 617 | 34.7  [22.9-50.5] | 27 /  778 617 | 34.7  [22.9-50.5] |
| **6 years** | 108 /  3 762 622  (714 482; 785 922) | 28.7  [23.5-34.7] | 152 /  3 469 256  (680 978; 707 735) | 43.8  [37.1-51.4] | 154 /  3 608 051  (705 448; 740 352) | 42.7  [36.2-50.0] | 34 /  759 271 | 44.8  [31.0-62.6] | 29 /  759 271 | 38.2  [25.6-54.9] | 29 /  759 271 | 38.2  [25.6-54.9] |

^1^Defined using group I(a)1 of the International Classification of Childhood Cancer, third edition (ICCC-3).

^2^Age-specific incidence rate per 1 000 000 person-years in 2015-2019. Incidence rates for 2015-2019 included all cases reported in the respective year or the subsequent year; cases reported only after the subsequent calendar year were not included.

^3^Age-specific incidence rate per 1 000 000 person-years in 2020. Incidence rates included all cases reported in the respective year or the subsequent year; cases reported only after the subsequent calendar year were not included.

^4^Although the GCCR receives information on newly diagnosed cases on a daily basis, some cases (5.8% of all BCP-ALL cases) of a calendar year are reported with some delay in the subsequent calendar year. For 2020, approximately 99.4% of all incident BCP-ALL cases of that year were reported by 15 March 2021. ASR for 2021 were estimated by applying two different hypothetical late-reporting scenarios.

^5^Scenario I: considering no additional cases due to late reporting after 15 March 2022.

^6^Scenario II: estimated age-specific incidence rate per 1 000 000 person-years in 2021, applying the proportion of additional cases due to late reporting after 15 March 2021 observed for incident diagnoses in 2020 (specific to single years of age). The proportion of additional cases due to late reporting after the 15 March 2021 amounted to 1.33% for the 2-year olds; for the other single years of age no additional cases have been reported.

^7^Minimum and maximum number of individuals at risk during the respective period
